# Comprehensive profiling of sulfated phenolic compounds in edible and infesting seaweeds by a dedicated software-assisted platform

**DOI:** 10.1007/s00216-025-06027-3

**Published:** 2025-08-28

**Authors:** Enrico Taglioni, Chiara Cavaliere, Andrea Cerrato, Aldo Laganà, Carmela Maria Montone, Anna Laura Capriotti

**Affiliations:** Department of Chemistry, Sapienza University of Rome, Piazzale Aldo Moro 5, 00185 Rome, Italy

**Keywords:** Algae, High-resolution mass spectrometry, Polyphenols, Sulfated phenolic acids, Compound Discoverer, Untargeted mass spectrometry

## Abstract

Seaweeds, comprising green (Chlorophyta), brown (Phaeophyta), and red (Rhodophyta) algae, are recognized as valuable marine resources rich in bioactive phenolic compounds with nutraceutical potential. Despite growing interest in edible species, limited attention has been given to infesting seaweeds, which may represent an underexploited source of bioactives while addressing ecological challenges. In the present study, an innovative, structure-based data processing workflow was set up for the characterization of phenolic compounds in marine organisms and applied to eight edible and two infesting seaweed species from the major taxonomic groups by means of untargeted mass spectrometric data acquisition. The results reveal distinct phenolic profiles across seaweed taxa, with green algae exhibiting the highest abundances of phenol and phenolic acid sulfates, while brown algae were characterized by a predominance of phlorotannins. Sulfated phenolics outnumbered their non-sulfated counterparts, underscoring the importance of dedicated analytical workflows for marine-specific metabolites. Multivariate statistical analyses further distinguished seaweed classes based on phenolic content and compound classes, with infesting seaweeds demonstrating comparable phenolic profiles to their edible counterparts. This suggests the potential for valorizing infesting seaweeds as sustainable sources of bioactive compounds, aligning with Blue Economy principles. Our findings provide comprehensive insights into the chemical diversity of seaweed phenolics and highlight the unexplored potential of invasive species for nutraceutical applications, contributing to sustainable marine resource management and the development of functional foods.

**Graphical Abstract:**

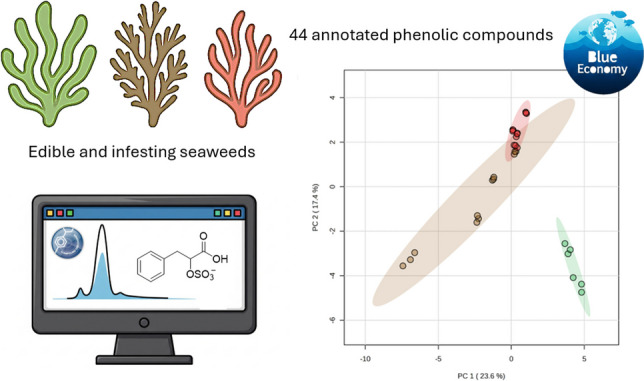

**Supplementary Information:**

The online version contains supplementary material available at 10.1007/s00216-025-06027-3.

## Introduction

Seaweeds constitute a taxonomically and morphologically diverse group of multicellular, photosynthetic organisms found in aquatic environments. Comprising thousands of species, they are broadly divided into three main taxonomic groups based on the dominant pigments present in their thallus: Chlorophyta (green algae), Phaeophyceae (brown algae), and Rhodophyta (red algae) [1]. Growing awareness among consumers, fueled by increasing concerns over the use of synthetic chemical additives, has recently contributed to a global shift in consumption habits [2]. This has led to a rising demand for natural-based products, prompting the food industry to adapt its strategies accordingly. In response, significant efforts have been directed toward identifying and utilizing natural alternatives that can both replace traditional synthetic ingredients and offer additional health benefits. These health-promoting compounds, widely known as nutraceuticals, have become central to the formulation of functional food [3]. Among the most explored sources of such compounds are seaweeds, which have long been recognized for their richness in bioactive molecules and their potential to enhance both food quality and human well-being [4]. Edible seaweed not only serves as a direct food item but also functions as dietary supplements, offering a rich profile of health-promoting nutrients. These include essential minerals, naturally occurring vitamins, polysaccharides, pigments, polyphenols, proteins, and peptides—compounds that have been extensively studied and shown to exhibit a range of beneficial biological activities, including antioxidant and antimicrobial effects [1, 5–9]. Nevertheless, algae are gaining significant recognition not only for their multifunctional properties as nutritious food alternatives or supplements but also as contributors to climate change mitigation through the provision of vital ecosystem services. They represent marine resources with significant potential to fulfill the purpose of the Sustainable Blue Economy [10], which extends the Circular Economy principles of sustainability and reuse to activities impacting the world's aquatic ecosystems. Indeed, the uncontrolled growth of algae in certain areas, leading to algal blooms, has raised concerns regarding their ecological impact on marine habitats and water quality [11]. Invasive seaweed can significantly disrupt the stability of aquatic ecosystems, notably through the accumulation of large quantities of wrack onshore. Unfortunately, less attention has been given to invasive seaweeds despite the several macroalgal taxa present in Italy [12]. Collecting and valorizing accumulated biomass represents a sustainable solution to this issue by contributing to the reduction of nutrient concentrations in marine environments and rebalancing the nitrogen-to-phosphorus ratio, which are key factors in controlling eutrophication. Interestingly, algae represent phylogenetic ancestors of land plants characterized by both primary metabolisms, devoted to life maintenance, cell structure, nutrition, and reproduction, and secondary metabolisms, which are fundamental for defense and adaptation to environmental conditions and threats [2]. Sulfated secondary metabolites comprise a diverse, and not fully explored, group of compounds that include glucosinolates, phytosulfokines, and other sulfated derivatives. Among this compound class, sulfated phenolic derivatives are commonly found in marine organisms [13]. However, the function of sulfation in plants has not been well clarified. Recent studies have highlighted that sulfated metabolites function as inactivated precursors to more active forms with diverse ecological roles that are released by sulfatases [14]. Sulfation enhances the hydrophilicity and solubility of metabolites, thereby increasing their bioavailability. It may also influence the pigmentation of seaweed biomass by stabilizing complexes with other chromophores. Moreover, it is hypothesized to play a role in the neutralization of toxic compounds, in the modulation of plant growth processes, and in participating in defense reactions against mechanical damage to the alga [15]. Although it is well known that edible seaweeds are rich in bioactive compounds, less information is available concerning infesting seaweeds. Even though the overall phenolic content in algae might be relatively low compared to some other organisms, they have been attracting a lot of attention since they represent an innovative source of antioxidants [2]. In this study, a comparison between commercial and infesting seaweeds was carried out regarding bioactive phenolic compounds to highlight specific composition differences and valorize a marine resource to which an environmental issue is related. Extracts derived from eight edible and two infesting seaweeds from the three main seaweed families (Ochrophyta, Chlorophyta, and Rhodophyta) were in-depth investigated by ultra-high-performance liquid chromatography coupled to high-resolution mass spectrometry (UHPLC-HRMS) followed by accurate data analysis and compound annotation employing an innovative structure-based workflow that was specifically dedicated to uncover novel sulfated metabolites. Afterward, a comparison of infesting and commercial seaweeds by using multivariate chemometric models in terms of bioactive compounds was carried out to gather comprehensive knowledge on the phenolic compound content of seaweeds from different taxa and to evaluate the nutraceutical potential of the infesting seaweed.

## Materials and methods

### Samples

Eight edible seaweed samples were purchased from Consonni Bioalghe Srls (Milan, Italy). Four Heterokontophyta (brown algae) of the class Phaeophyceae were analyzed, i.e., *Laminaria digitata* (kombu), *Saccharina latissima* (sugar kelp), *Himanthalia elongata* (sea spaghetti), and *Undaria pinnatifida* (wakame), as well as one Chlorophyta (green algae) of the family Ulvophyceae, i.e., *Ulva lactuca* (sea lettuce), and three Rhodophyta (red algae) of the class Florideophyceae, i.e., *Chondrus crispus* (Irish moss) and *Palmaria palmata* (dulse), and Bangiophyceae, i.e., *Porphyra umbilicalis* (Atlantic nori). *Gracilaria gracilis* (Florideophyceae) and *Ulva Rigida* (Ulvophyceae) infesting algae were collected in the northern coastal lagoon. Sampling sites were selected based on the presence of *Gracilaria spp.* and the occurrence of algal infestations. The sampling was conducted onshore (on land) and offshore (open sea). Field surveys were performed during May–June 2024 to coincide with peak infestation periods. Infested seaweed specimens were randomly collected from intertidal and shallow subtidal zones at each site using handpicking and wading techniques. A minimum of 10 samples were collected per site to ensure representative sampling. Collected samples were placed in labeled zip-lock plastic bags containing seawater to prevent desiccation. Samples were transported to the laboratory in an icebox at 4 °C to minimize degradation. Upon arrival, samples were gently rinsed with filtered seawater to remove debris and epiphytes. Samples were freeze-dried by a Heto PowerDry LL1500 (Thermo Fisher), finely ground in a mortar, and stored at − 20 °C until use.

### Chemicals

Optima® LC–MS grade water, methanol (MeOH), and acetonitrile (ACN) were purchased from Thermo Fisher Scientific (Waltham, MA, USA). Acetone, acetic acid, and formic acid were purchased from Merck (Kenilworth, NJ, USA). Apigenin, apigenin 7-*O*-glucoside, biochanin A, 3-caffeoylquinic acid, caffeic acid, diosmetin, epicatechin, eriodyctiol, ferulic acid, hesperetin, kaempferol, luteolin, myricetin, naringenin, p-coumaric acid, procyanidin B1, procyanidin B2, rutin, quercetin, quercetin 3-*O*-glucoside, and taxifolin analytical standards were purchased from Merck. A phenolic compound working mix solution was prepared at 0.5 μg mL^−1^ in H_2_O/MeOH 90:10 (*v/v*), aliquoted, and stored at − 20 °C for further use.

### Seaweed phenolic compounds extraction

Phenolic compounds were extracted following an established protocol that was previously described [16] Briefly, 200 mg of freeze-dried seaweed sample was extracted with 4 mL CH_3_COCH_3_/H_2_O/CH_3_COOH (70:29.5:0.5, *v/v/v*). The extract was sonicated for 15 min in an ice bath and then centrifuged for 10 min at 2000 × g. The supernatant was collected, and the procedure was repeated once. The supernatants were mixed and concentrated to 900 µL using a Speed-Vac SC 250 Express (Thermo 164 Avant, Holbrook, NY, USA). Then, 100 μL of MeOH was added to the sample, and the final extract solution (H_2_O/MeOH, 90:10 *v/v*) was filtered through a 13-mm Acrodisc Syringe filter with a 0.2 μm GH Polypro membrane (Pall, Ann Arbor, MI, USA). Finally, the extract was aliquoted and stored at − 20 °C for further analysis.

### UHPLC-HRMS analysis

A Vanquish binary pump H (Thermo Fisher Scientific, Bremen, Germany), equipped with a thermostated autosampler and column compartment, was used for polyphenol chromatographic separation on a Kinetex XB C18 column (100 mm × 2.1 mm i.d.) with a particle size of 2.6 μm (Phenomenex, Torrance, CA, USA) at 40 °C and with a flow rate of 600 μL min^−1^ as described in a previous work [16]. The UHPLC system was coupled to a Q Exactive hybrid quadrupole-Orbitrap mass spectrometer (Thermo Fisher Scientific) with a heated ESI source operated in negative ion mode (ESI-). The ESI source and MS parameters were set as reported in our previous work [16]All samples were run in triplicate, followed by the injection of the standard mix and a blank sample of H_2_O/MeOH (90:10, *v/v*). The injection volume was 10 μL. Raw MS/MS data files were acquired by Xcalibur software (version 3.1, Thermo Fisher Scientific). For system suitability testing, the column stability and performance were tested before and after each analytical section using solvent blank samples (H_2_O/MeOH, 90:10, *v/v*) and working mix standard solutions.

### Sulfated phenolic database compilation

The molecular formulas and molecular weights of 32 phenolic compound non-sulfated aglycones were taken into consideration, including phenols (i.e., phenol, catechol, phloroglucinol, vanillin, tyrosol, and hydroxytyrosol), phenolic acids (i.e., benzoic acid, hydroxybenzoic acid, dihydroxybenzoic acid, gallic acid, vanillic acid, coumaric acid, caffeic acid, phenyllactic acid, hydroxyphenyllactic acid, syringic acid, ferulic acid, hydroxyferulic acid, sinapic acid, hydroxynaphtoic acid, dihydroxhynaphtoic acid, and ellagic acid), coumarins (i.e., hydroxycoumarin, dihydroxycoumarin, scopoletin, and scoparone), and phlorotannins (i.e., eckol dimer, eckol, diphloroethol, triphloroethol, tetraphloroethol, bifuhalol). The molecular formulas and molecular weights of the selected non-sulfated aglycones were then combined using Excel with one of four sugar/sugar-like moieties (hexose, pentose, quinic acid, and shikimic acid) to generate 160 non-sulfated phenolic compounds, that were finally combined with one, two, or three sulfated groups, resulting in a total of 640 sulfated and non-sulfated phenolic compounds.

### Data processing and compound annotation

The.raw data obtained by the analysis of the extracted seaweeds and the process blank were preprocessed using the software Compound Discoverer version 3.1 (Thermo Fisher Scientific) using a dedicated data processing workflow that was set up for the annotation of sulfated and non-sulfated phenolics. In particular, the customized workflow was created using the same rationale as previously reported [16]. Data preprocessing allowed feature alignment, background removal, adduct definition and grouping, and molecular formula annotation. Moreover, the phenolic compound database described in the “Sulfated phenolic database compilation” section was employed as a Mass List, thus allowing the automatic match of the experimental molecular weights and formulas with the computed ones and filtering out the non-matching features. MS/MS spectra of the filtered features were finally manually validated to assign the tentative identification according to the typical fragmentation pathways of phenolic compounds.

### Statistical analysis

MetaboAnalyst 6.0 was employed for statistical analysis and data visualization [17]. Following the specific indications furnished by the developers, the data matrix was submitted as a comma-separated value file. The interquartile range (IQR) was selected for data filtering, whereas the autoscaling algorithm was selected for data scaling. The phenolic compound data matrices were submitted to MetaboAnalyst for hierarchical clustering information (dendrogram and heatmap), principal component analysis (PCA), and correlation heatmaps.

## Results and discussion

### Sulfated and non-sulfated phenolic compound annotation

For the profiling of phenolic phytocompounds, the seaweed samples were extracted using a common solvent mixture (70% acetone) that has been employed for a variety of different plant or plant-like materials, such as herbs and leafy vegetables [18], fruits [19, 20], berries [21, 22], flowers [16, 23], and seaweeds [24]. The seaweed extracts were then analyzed by LC–MS/MS in an untargeted fashion, using a stepped NCE fragmentation method that ensures richer and more diagnostic MS/MS spectra, especially in the case of compounds conjugated with sugar via acetal bonds [16, 25]. In particular, the acquisition was performed with a three-step normalized collision energy of 20–40–60 NCE, thus allowing simultaneous fragmentation of weak acetal bonds and strong aromatic and/or conjugated aglycones. The untargeted approach was aimed at gathering comprehensive knowledge on the phenolic compound composition of edible seaweeds, which has not been fully explored yet [9]. Seaweeds are known to display a unique composition of phenolics, which has recently gained attention for the numerous beneficial effects on the human body through seaweed consumption [26, 27]. In particular, sulfated phenolics are believed to be solely produced by marine organisms, whereas other sulfur-containing compounds, such as glucosinolates, are synthesized by terrestrial plants [28].

For these reasons, the untargeted datasets were first processed using a previously set up data analysis platform that was customized on Compound Discoverer software for phenolic compounds [16]. The data processing platform was based on a suspect screening compound annotation approach, in which a comprehensive mass list of 45,567 phenolic compound derivatives, including flavonoids, anthocyanins, phenolic acids, ellagitannins, and proanthocyanidins, either free or conjugated to sugars, cyclitols, and carboxylic acids, was created. These preliminary experiments were aimed at screening the nature of the non-sulfated phenolics to build a phenolic compound database for seaweeds that would also consider the sulfation of the free hydroxyl groups. Unsurprisingly, phenolic acids of various subclasses were annotated, including hydroxybenzoic acids (e.g., dihydroxybenzoic acid), hydroxycinnamic acids (e.g., coumaric acid), and hydroxyphenylacetic acids (e.g., phenyllactic acid). Conversely, none of the flavonoid/anthocyanin — either free or conjugated — entries in the database were matched. For these reasons, and with the support of the literature [9], a database of sulfated and non-sulfated phenols, phenolic acids, coumarins, and phlorotannins was built as described in the “Materials and methods” section and was employed as a Mass List for a new customized data processing workflow on Compound Discoverer.

Following data preprocessing, a list of 53,429 features was extracted from the seaweed datasets. However, data filtering based on the customized Mass List allowed the removal of 53,068 features, whose molecular formulas and weights were not present in the datasets and whose MS spectrum was not associated with an MS2 spectrum. The dedicated data processing workflow therefore filtered out more than 99% of the original features, thus leaving only 361 features for manual validation of the MS data. Following careful spectral annotation, 44 phenolics were tentatively identified, as reported in Table 1 with their identification data, i.e., retention time (RT), molecular formula, molecular weight, experimental m/z, mass error, diagnostic production ions, and confidence level according to Schymanski et al. [29].

**Table 1:** Retention times (RT, min), proposed formulas, experimental m/z, accuracy (Δ, ppm), main diagnostic product ions, and confidence level of the identification (C. L.) of the 44 annotated phenolic compounds in edible and infesting seaweeds

ID	Compound	RT	Molecular formula	Molecular weight	Experimental m/z	Δmass	Diagnostic product ions	C.L
1	Phloroglucinol sulfate	0.6	C_6_H_6_O_6_S	205.9883	204.9811	− 0.8	204.9813; 125.0243; 79.9569	2
2	Diphloroethol disulfate	0.7	C_12_H_10_O_12_S_2_	409.9613	408.9540	− 0.2	328.9972; 249.0403; 141.0193; 140.0114; 125.0243; 79.9565	2
3	Diphloroethol trisulfate	0.7	C_12_H_10_O_15_S_3_	489.9188	488.9115	1.2	408.9536; 328.9972; 249.0403; 141.0193; 140.0114; 125.0243; 79.9565	2
4	Catechol sulfate	0.8	C_6_H_6_O_5_S	189.9932	188.9859	− 2.4	188.9855; 109.0289; 79.9565	3
5	Diphloroethol sulfate	0.8	C_12_H_10_O_9_S	330.0045	328.9972	− 0.2	328.9972; 249.0403; 141.0193; 140.0114; 125.0243	2
6	Diphloroethol	0.8	C_12_H_10_O_6_	250.0477	249.0404	− 0.1	249.0400; 141.0193; 125.0243	2
7	Hydroxymethoxybenzoic acid sulfate	0.9	C_8_H_8_O_7_S	247.9983	246.9911	− 3.0	246.9907; 203.0012; 123.0290; 108.0211; 80.9644; 79.9566	3
8	Dihydroxyphenyllactic sulfate 1	1.0	C_9_H_10_O_8_S	278.0087	277.0014	− 3.5	277.0014; 197.0449; 179.0340; 135.0445; 96.9596; 72.9922	3
9	Dihydroxyphenyllactic disulfate	1.0	C_9_H_10_O_11_S_2_	357.9652	356.9580	− 3.4	277.0014; 197.0449; 179.0340; 135.0445; 96.9596; 79.9565; 72.9922	2
10	Dihydroxybenzoic acid	1.3	C_7_H_6_O_4_	154.0264	153.0191	− 1.6	153.0188; 109.0291	2
11	Dihydroxyphenyllactic sulfate 2	1.3	C_9_H_10_O_8_S	278.0092	277.0019	− 1.7	277.0014; 197.0449; 179.0340; 135.0445; 123.0447; 79.9565; 72.9922	3
12	Dihydroxybenzoic acid sulfate	1.3	C_7_H_6_O_7_S	233.9830	232.9757	− 1.9	232.9753; 153.0188; 109.0291	2
13	Hydroxybenzaldehyde isomer sulfate	1.4	C_7_H_6_O_5_S	201.9928	200.9855	− 4.0	200.9864; 121.0289; 79.9565	3
14	Triphloroethol	1.4	C_18_H_14_O_9_	374.0638	373.0566	0.1	373.0574; 247.0244; 229.0138; 201.0190; 125.0242	2
15	Hydroxybenzoic acid sulfate	1.4	C_7_H_6_O_6_S	217.9876	216.9803	− 4.2	216.9802; 137.0238; 93.0339	2
16	Phenol sulfate	1.5	C_6_H_6_O_4_S	173.9980	172.9907	− 3.9	172.9906; 93.0340; 79.9566	2
17	Hydroxyphenyllactic acid sulfate	1.5	C_9_H_10_O_7_S	262.0137	261.0064	− 4.0	261.0064; 181.0505; 163.0399; 135.0451; 119.0501; 79.9565	2
18	Triphloroethol sulfate	1.5	C_18_H_14_O_12_S	454.0210	453.0137	0.8	453.0130; 373.0574; 247.0244; 229.0138; 201.0190; 125.0242; 79.9565;	2
19	Hydroxyphenyllactic acid	1.6	C_9_H_10_O_4_	182.0579	181.0506	− 0.3	181.0505; 163.0399; 135.0451; 119.0501; 101.0241; 89.0240	2
20	Hydroxytyrosol sulfate	1.7	C_8_H_10_O_6_S	234.0193	233.0120	− 2.3	233.0118; 153.0553; 123.0449; 79.9567	2
21	Dihydroxyphenylacetic acid	1.8	C_8_H_8_O_4_	168.0419	167.0346	− 2.5	167.0342; 123.0446	3
22	Dihydroxyphenylacetic acid sulfate	1.8	C_8_H_8_O_7_S	247.9988	246.9915	− 1.1	246.9909; 167.0342; 123.0446; 79.9567	2
23	Dihydroxycoumarin 1	1.9	C_9_H_6_O_4_	178.0261	177.0188	− 3.2	177.0184; 133.0292	3
24	Hydroxyphenylacetic acid sulfate	2.0	C_8_H_8_O_6_S	232.0031	230.9958	− 4.6	230.9960; 151.0396; 107.0499; 79.9567	2
25	Tyrosol sulfate	2.0	C_8_H_10_O_5_S	218.0252	217.0179	1.4	217.0164; 137.0607; 79.9568	2
26	Hydroxyphenylacetic acid	2.0	C_8_H_8_O_3_	152.0473	151.0400	− 0.5	151.0396; 107.0499	2
27	Hydroxymethoxyphenylacetic acid sulfate	2.3	C_9_H_10_O_7_S	262.0141	261.0068	− 2.6	261.0064; 181.0505; 122.02679	3
28	Hydroxybenzaldehyde sulfate	2.4	C_7_H_6_O_5_S	201.9932	200.9859	− 2.0	200.9864; 121.0289; 93.0343; 79.9565	2
29	Dihydroxybenzaldehyde sulfate	2.7	C_7_H_6_O_6_S	217.9875	216.9802	− 4.6	216.9801; 137.0237; 109.0291; 108.0211; 80.9643; 79.9565	2
30	Caffeic acid	3.5	C_9_H_8_O_4_	180.0419	179.0347	− 1.8	179.0345; 135.0448	2
31	Dihydroxymethylcoumarin	3.9	C_10_H_8_O_4_	192.0418	191.0346	− 2.3	191.0341; 147.0444; 93.0338	3
32	Phloretic acid sulfate	4.2	C_9_H_10_O_6_S	246.0197	245.0124	− 0.5	245.0124; 165.0548; 121.0658; 93.0342 79.9569	3
33	Caffeic acid isomer 1	4.2	C_9_H_8_O_4_	180.0417	179.0345	− 2.9	179.0343; 135.0448; 109.0291	3
34	Phenyllactic acid sulfate	4.9	C_9_H_10_O_6_S	246.0187	245.0114	− 4.7	165.0555; 147.0451; 119.0501; 96.9595; 72.9926	2
35	Phenyllactic acid	5.3	C_9_H_10_O_3_	166.0627	165.0555	− 1.5	165.0555; 147.0451; 119.0501; 72.9926	2
36	Dihydroxycoumarin 2	5.3	C_9_H_6_O_4_	178.0261	177.0188	− 3.1	177.0184; 133.0292	3
37	Coumaric acid	5.8	C_9_H_8_O_3_	164.0468	163.0395	− 3.6	163.0391; 119.0496	2
38	Caffeic acid isomer 2	6.2	C_9_H_8_O_4_	180.0418	179.0345	− 2.6	179.0343; 135.0448; 109.0291	3
39	Phloretic acid isomer 1	6.3	C_9_H_10_O_3_	166.0627	165.0554	− 1.7	165.0548; 121.0658; 93.0342	3
40	Phloretic acid isomer 2	6.7	C_9_H_10_O_3_	166.0626	165.0553	− 2.3	165.0548; 121.0658; 93.0342	3
41	Dihydroxydimethylcoumarin 1	7.2	C_11_H_10_O_4_	206.0576	205.0503	− 1.6	205.0501; 161.0602; 93.0340; 75.0080	3
42	Phloretic acid isomer 3	7.3	C_9_H_10_O_3_	166.0624	165.0551	− 3.8	165.0548; 121.0658; 95.0495; 93.0342	3
43	Dihydroxydimethylcoumarin 2	7.7	C_11_H_10_O_4_	206.0577	205.0504	− 1.2	205.0501; 161.0602; 133.0656	3
44	Dihydroxydimethylcoumarin sulfate	8.6	C_11_H_10_O_7_S	286.0149	285.0077	0.7	295.0075; 205.0501; 161.0602; 133.0656	3

The largest group of annotated phenolics was phenolic acids (24 identifications), equally distributed between sulfated and non-sulfated compounds, followed by phenol sulfates (8 compounds), phlorotannins, and coumarins (6 compounds each). Overall, sulfated phenolic compounds outnumbered their non-sulfated counterparts (25 and 19 annotations, respectively), thus confirming the need for a dedicated data processing platform for the annotation of such unusual phenolic structures when seaweed samples are characterized.

Retention times were helpful for compound annotation. Homolog structures that differ for the number of hydroxyl groups were eluted from the most to the least hydrolyzed, e.g., phloroglucinol sulfate (compound 1; two free OH groups, RT = 0.6) < catechol sulfate (compound 4; one free OH groups, RT = 0.8) < phenol sulfate (compound 16; no free OH groups, RT = 1.5). Moreover, the introduction of a sulfate group generally determined a slightly lower RT compared to the non-sulfated counterpart, e.g., phenyllactic acid sulfate (compound 34, RT = 4.9) < phenyllactic acid (compound 35, RT = 53). One exception to these trends regards compounds 41 and 43, annotated as two isomers of dihydroxydimethyl coumarin, which eluted earlier than compound 44, which was attributed to dihydroxydimethyl coumarin sulfate. However, coumarin peaks had an extremely low intensity in all analyzed seaweeds, and there were other peaks with the same m/z as compounds 41 and 43 at higher retention times that could not be annotated due to their even lower ion intensity. Compound 44 might effectively be a sulfated derivative of one of these possible unidentified peaks.

**Fig. 1:**
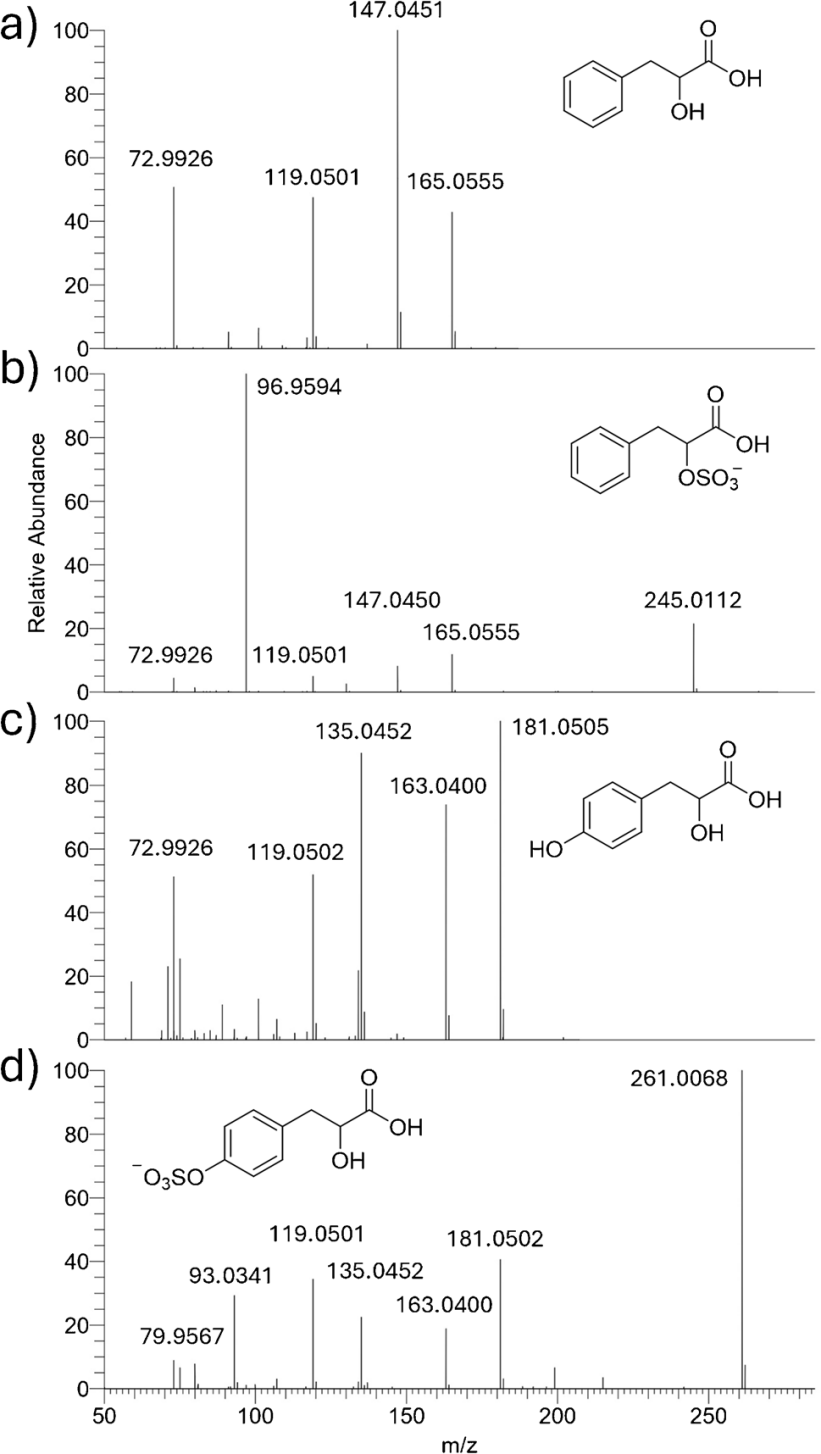
Tandem MS spectra of annotated phenyllactic acid (**a**, RT = 1.6), phenyllactic acid sulfate (**b**, RT = 1.5), hydroxyphenyllactic acid (**c**, RT = 5.3), and hydroxyphenyllactic acid sulfate (**d**, RT = 4.9) from the seaweed extract datasets

Figure 1 shows the MS/MS of two phenolic acid sulfates in comparison with their non-sulfated counterparts. Phenyllactic acid (compound 35, Fig. 1a) is an important broad-spectrum antimicrobial compound [30], whose identification was based on the match of the experimental MS/MS spectrum to the one in the mzCloud database (https://www.mzcloud.org/). Its sulfated counterpart (compound 34, Fig. 1b) was annotated based on the presence of the three main diagnostic ions of phenyllactic acid (m/z 165.0555, 147.0450, and 119.0501) as well as an intense ion at m/z 96.9594, which corresponds to HSO_4_^−^, thus indicating the sulfation of the alkylic OH group of phenyllactic acid. Hydroxyphenyllactic acid (compound 19, Fig. 1c) was again annotated based on the match of the experimental MS/MS spectrum to the mzCloud database and shows analog fragmentation as phenyllactic acid, with sequential H_2_O and CO neutral losses (m/z 163.0400 and 135.0452). Moreover, the aromatic OH group stabilizes the negative charge and allows the cleavage of the carboxyl group (m/z 119.0502). Similarly, all the diagnostic product ions of hydroxyphenyllactic acid were present in the MS/MS spectrum of the sulfated analog (compound 17, Fig. 1d). However, the peak of the sulfate group was found at m/z 79.9567 (instead of m/z 96.9594), thus indicating the location of the sulfation. The bond dissociation energies of C–OH bonds for methanol and phenol are estimated at around 90 and 110 kcal mol^−1^, respectively [31]. As such, due to the significantly higher bond dissociation energy (BDE) of C–OH bonds of phenol groups, the loss of sulfate groups located onto aromatic rings generates an SO_3_^−^ ion rather than the HSO_4_^−^, indicating cleavage of the O-S bond rather than the C-O bond. These results are in agreement with the MS/MS spectra of phenol and catechol sulfates, which possess only aromatic OH groups and only generate SO_3_^−^ ions. The same rationale applies to hydroxybenzoic acid sulfate (compound 15), dihydroxybenzoic acid sulfate (compound 12), hydroxyphenylacetic acid sulfate (compound 24), dihydroxyphenylacetic acid sulfate (compound 22), and hydroxymethoxyphenylacetic acid sulfate (compound 27), whose only possible site of sulfation is the aromatic OH group. Interestingly, the two isomers of dihydroxyphenyllactic sulfate could be distinguished based on the site of the sulfation. The first isomer (compound 8) generated an intense HSO_4_^−^ ion typical of the sulfation of the benzyl alcohol, whereas the sulfate group was located on one of the phenol groups on the second isomer (compound 11). Finally, the disulfate of dihydroxyphenyllactic acid (compound 9) showed both diagnostic ions, thus indicating the sulfation of both the benzyl and the phenyl OH groups. Despite the coexistence of aromatic and aliphatic OH groups, the sulfation of tyrosol and hydroxytyrosol only occurred on the phenol rings (compounds 25 and 20).

### Phenolic compound content in edible and infesting seaweeds

**Fig. 2:**
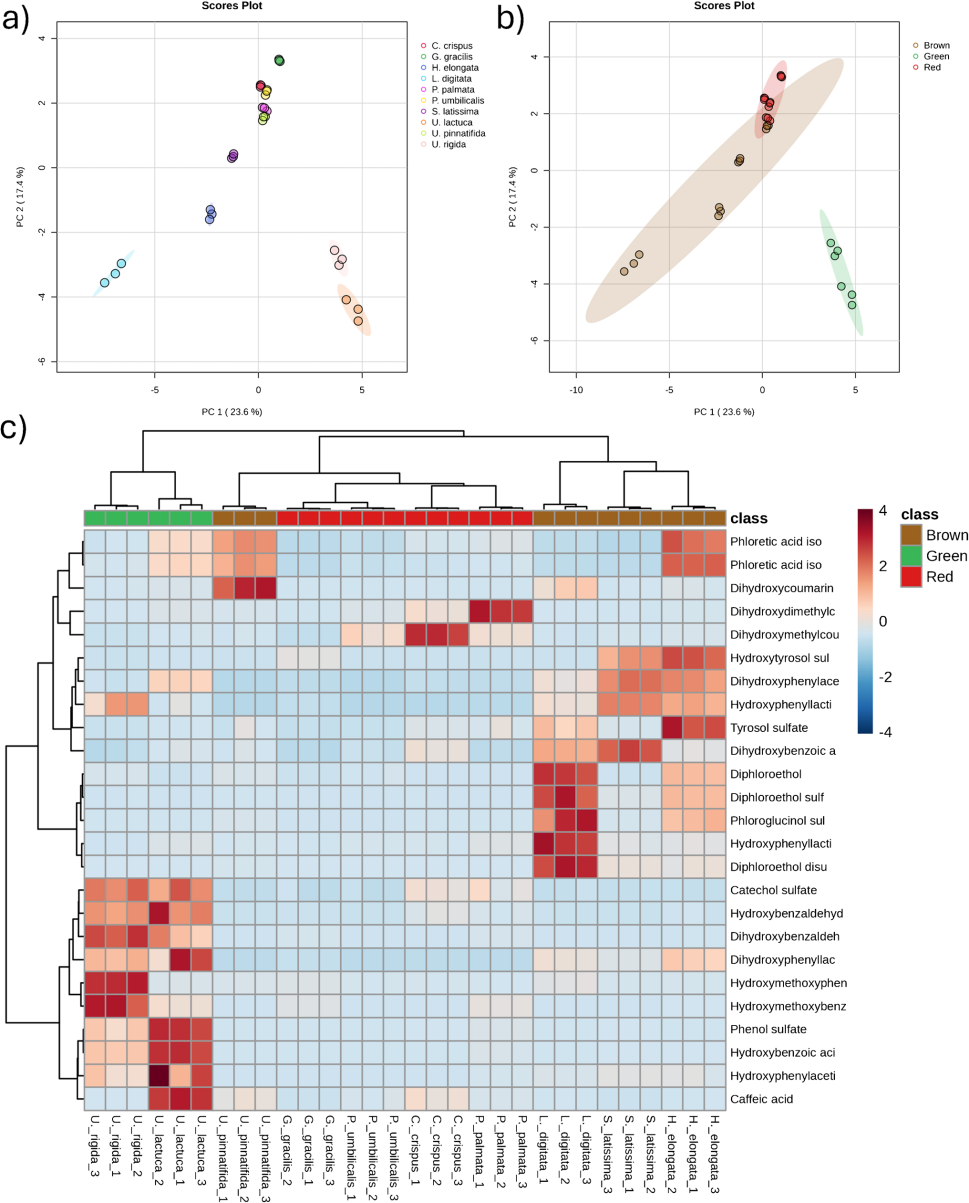
Scores plots of the principal component analysis using the 44 annotated phenolic compounds in the 10 seaweed strains analyzed individually (**a**) and grouped based on class (**b**). Hierarchical cluster dendrogram and heatmap using the 44 annotated phenolic compounds in the three groups of seaweeds (**c**)

The annotated sulfated and non-sulfated phenolics data matrix was subjected to statistical analysis to assess possible trends and variability in the content of phenolic compounds and compound classes in the distinct analyzed samples. Metaboanalyst was employed for data processing [17], and four distinct analyses were carried out: (i) the content of the single annotated compounds in the ten seaweed samples, (ii) the content of the single annotated compounds in the three seaweed classes (brown vs red vs green), (iii) the content of the phenol compound classes in the ten seaweed samples, and (iv) the content of the phenol compound classes in the three seaweed classes. The 44 annotated compounds were grouped into five classes: phenol sulfates (7 compounds), phenolic acids (12 compounds), phenolic acid sulfates (12 compounds), coumarins (6 compounds), and phlorotannins (7 compounds). Sulfated and non-sulfated coumarins and phlorotannins had to be grouped due to their low number. Preliminary studies were conducted to determine the distribution of the annotated compounds in the six subclasses. In particular, the correlation heatmap hierarchical clustering shown in Figure S1 was employed to allocate phloroglucinol sulfate in the phlorotannin sulfate subclass. Despite being structurally a phenol sulfate, phloroglucinol sulfate was highly correlated with phlorotannin sulfates, for which phloroglucinol is a monomer. Figure 1a shows the principal component analysis (PCA) based on the content of the single annotated compounds in the ten seaweed samples. Alongside principal component 1 (PC1), explaining 23.6% of the total variance, the two analyzed green seaweeds (*U. lactuca* and *U. rigida*) were clustered at positive values, whereas three brown seaweeds (*L. digitata*, *H. elongata*, and *S. latissima*) had negative values. The other five samples (all red seaweeds and *U. pinnatifida*) were clustered around zero. Figure 1b shows the same data when seaweeds are grouped based on their taxonomy. Both red and green seaweeds were highly clustered, whereas the brown algae were more dispersed, with Wakame algae (*U. pinnatifida*) overlapping red algae and being at a long distance from *L. digitata*. Green algae showed a generally higher abundance of phenol and phenolic acid sulfates, as shown in Figures S2a and S2b, for phenol sulfate and catechol sulfate, respectively. Conversely, brown seaweeds — with the exception of Wakame — were characterized by phlorotannin sulfates, as shown in Figures S2c-f. In particular, the results shown in Figure S2c for phloroglucinol sulfate confirm the correlation with phlorotannins discussed earlier. Phlorotannins are known to be uniquely produced by brown seaweeds and have gained interest for their antioxidant, anti-inflammatory, anti-cancer, and antimicrobial properties [32]. The hierarchical cluster dendrogram and heatmap (Fig. 2c) summarize the results in terms of the content of the single annotated phenolic compounds in the analyzed seaweed samples. The dendrogram confirms that green algae stand out from both red and brown algae. Moreover, Wakame had a phenol profile that was much closer to the four analyzed red seaweeds compared to the other three brown algae, which had the highest abundance in terms of phlorotannins as well as tyrosol derivatives. It appears clear that, except for some coumarins, red algae had, in general, a lower phenol content. Interestingly, coumarins had relatively higher concentrations also in *U. pinnatifida*. These findings might be attributed to the convergent evolution of brown algae, which developed a plant-like body plan and specialized transport network despite being only distantly related to plants [33].

The content of the phenolic compound classes was then investigated. As shown in Figure S3, phenol sulfates and phenolic acid sulfates had their highest abundances in *U. lactuca* and *U. rigida*, whereas phlorotannins had their highest abundance in brown algae (except *U.pinnatifida*). The coumarin content was more variable, with the highest abundance in red *C. crispus* and *P. palmata* and brown *U. pinnatifida* and *H. elongata*, while the lowest areas were in the two analyzed green seaweeds and red *G. gracilis*. The latter three also shared similar high phenol sulfate content. The trends based on the algae class are shown in Figure S4, in which the specificity of phenolic acid sulfate and phlorotannins for green and brown seaweeds, respectively, is even more evident, as well as the inconsistent abundance of coumarins based on the seaweed taxonomy. Green algae had the highest total amount of phenolic compounds based on the total peak area of the annotated compounds, as seen in Figure S3–4, in which the areas of phenol sulfates and phenolic acid sulfates were up to three orders of magnitude higher than in brown and red seaweeds.

A significant result that can be inferred from the experimental data is related to the comparison between edible and infesting seaweeds. In terms of the phenolic compound content, there seem to be no substantial differences between edible and infesting seaweeds belonging to the same taxon. Purchased edible *U. lactuca* and infesting *U. rigida* from the Adriatic Sea were effectively highly correlated, both rich in sulfated phenols and phenolic acids. These results hint at the possible use of infesting seaweed as a source of bioactive compounds in line with the principles of the Blue Economy.

## Conclusions

This study presents a comprehensive untargeted LC–MS/MS profiling of phenolic compounds in seaweed species from different taxonomic classes (brown, red, and green algae). For the first time, a dedicated data analysis platform was set up for the characterization of phenolic compounds in marine organisms, with a specific focus on sulfated phenolics. The data revealed a distinctive distribution of phenolic compounds across seaweed classes, with green algae exhibiting significantly higher levels of phenol sulfates and phenolic acid sulfates, while brown algae are characterized by abundant phlorotannins and their sulfated derivatives. A key finding is the close similarity in phenolic profiles between edible and infesting seaweeds within the same taxonomic groups, especially between *U. lactuca* (edible) and *U. rigida* (infesting). This suggests that infesting seaweeds, often considered a nuisance, may serve as an untapped resource for bioactive compounds with potential applications in nutrition, pharmaceuticals, and the Blue Economy.

Overall, this work contributes valuable insight into the chemical diversity of seaweed phenolics and provides a robust methodological framework for future studies aiming to explore marine natural products and the sustainable exploitation of seaweed biomass.

## Supplementary Information

Below is the link to the electronic supplementary material.

Supplementary Material 1 (DOCX 1.06 MB)

## Data Availability

Data are available from the authors by request.

## References

[1] Ramu Ganesan A, Subramani K, Shanmugam M, Seedevi P, Park S, Alfarhan AH, Rajagopal R, Balasubramanian B. A comparison of nutritional value of underexploited edible seaweeds with recommended dietary allowances. J King Saud Univ - Sci. 2020;32:1206–11. 10.1016/j.jksus.2019.11.009.

[2] Garcia-Perez P, Cassani L, Garcia-Oliveira P, Xiao J, Simal-Gandara J, Prieto MA, Lucini L. Algal nutraceuticals: A perspective on metabolic diversity, current food applications, and prospects in the field of metabolomics. Food Chem. 2023;409: 135295. 10.1016/j.foodchem.2022.135295. 36603477 10.1016/j.foodchem.2022.135295

[3] Vignesh A, Amal TC, Sarvalingam A, Vasanth K. A review on the influence of nutraceuticals and functional foods on health. Food Chem Adv. 2024;5: 100749. 10.1016/j.focha.2024.100749.

[4] Choudhary B, Chauhan OP, Mishra A. Edible seaweeds: a potential novel source of bioactive metabolites and nutraceuticals with human health benefits. Front Mar Sci. 2021;8. 10.3389/fmars.2021.740054.

[5] Škrovánková S. Seaweed vitamins as nutraceuticals. Adv Food Nutr Res. 2011;357–369. 10.1016/B978-0-12-387669-0.00028-4. 10.1016/B978-0-12-387669-0.00028-4 22054961

[6] Circuncisão A, Catarino M, Cardoso S, Silva A. Minerals from Macroalgae Origin: Health Benefits and Risks for Consumers. Mar Drugs. 2018;16:400. 10.3390/md16110400. 30360515 10.3390/md16110400 PMC6266857

[7] Zhong H, Gao X, Cheng C, Liu C, Wang Q, Han X. The Structural Characteristics of Seaweed Polysaccharides and Their Application in Gel Drug Delivery Systems. Mar Drugs. 2020;18:658. 10.3390/md18120658. 33371266 10.3390/md18120658 PMC7765921

[8] Aryee AN, Agyei D, Akanbi TO. Recovery and utilization of seaweed pigments in food processing. Curr Opin Food Sci. 2018;19:113–9. 10.1016/j.cofs.2018.03.013.

[9] Cotas J, Leandro A, Monteiro P, Pacheco D, Figueirinha A, Gonçalves AMM, da Silva GJ, Pereira L. Seaweed Phenolics: From Extraction to Applications. Mar Drugs. 2020;18:384. 10.3390/md18080384. 32722220 10.3390/md18080384 PMC7460554

[10] Armeli Minicante S, Bongiorni L, De Lazzari A. Bio-Based Products from Mediterranean Seaweeds: Italian Opportunities and Challenges for a Sustainable Blue Economy. Sustainability. 2022;14:5634. 10.3390/su14095634.

[11] Rudovica V, Rotter A, Gaudêncio SP, Novoveská L, Akgül F, Akslen-Hoel LK, Alexandrino DAM, Anne O, Arbidans L, Atanassova M, Bełdowska M, Bełdowski J, Bhatnagar A, Bikovens O, Bisters V, Carvalho MF, Catalá TS, Dubnika A, Erdoğan A, Ferrans L, Haznedaroglu BZ, Setyobudi RH, Graca B, Grinfelde I, Hogland W, Ioannou E, Jani Y, Kataržytė M, Kikionis S, Klun K, Kotta J, Kriipsalu M, Labidi J, Lukić Bilela L, Martínez-Sanz M, Oliveira J, Ozola-Davidane R, Pilecka-Ulcugaceva J, Pospiskova K, Rebours C, Roussis V, López-Rubio A, Safarik I, Schmieder F, Stankevica K, Tamm T, Tasdemir D, Torres C, Varese GC, Vincevica-Gaile Z, Zekker I, Burlakovs J. Valorization of Marine Waste: Use of Industrial By-Products and Beach Wrack Towards the Production of High Added-Value Products. Front Mar Sci. 2021;8. 10.3389/fmars.2021.723333.

[12] Petrocelli A, Cecere E. A 20-year update on the state of seaweed resources in Italy. Bot Mar. 2019;62:249–264 . 10.1515/bot-2018-0072.

[13] Gil-Martínez L, Santos-Mejías A, De la Torre-Ramírez JM, Baños A, Verardo V, Gómez-Caravaca AM. Optimization of a Sonotrode Extraction Method and New Insight of Phenolic Composition of Fucus vesiculosus. Mar Drugs. 2025;23:40. 10.3390/md23010040. 39852542 10.3390/md23010040 PMC11766535

[14] Supikova K, Kosinova A, Vavrusa M, Koplikova L, François A, Pospisil J, Zatloukal M, Wever R, Hartog A, Gruz J. Sulfated phenolic acids in plants. Planta. 2022;255:124. 10.1007/s00425-022-03902-6. 35562552 10.1007/s00425-022-03902-6

[15] Wekre ME, Holmelid B, Underhaug J, Pedersen B, Kopplin G, Jordheim M. Characterization of high value products in the side-stream of Laminaria hyperborea alginate production - Targeting the phenolic content. Algal Res. 2023;72: 103109. 10.1016/j.algal.2023.103109.

[16] Cerrato A, Cannazza G, Capriotti AL, Citti C, La Barbera G, Laganà A, Montone CM, Piovesana S, Cavaliere C. A new software-assisted analytical workflow based on high-resolution mass spectrometry for the systematic study of phenolic compounds in complex matrices. Talanta. 2020;209: 120573. 10.1016/j.talanta.2019.120573. 31892002 10.1016/j.talanta.2019.120573

[17] Xia J, Wishart DS. Metabolomic Data Processing, Analysis, and Interpretation Using MetaboAnalyst. Curr Protoc Bioinforma. 2011;34. 10.1002/0471250953.bi1410s34. 10.1002/0471250953.bi1410s34 21633943

[18] Sulaiman SF, Sajak AAB, Ooi KL, Supriatno SEM. Effect of solvents in extracting polyphenols and antioxidants of selected raw vegetables. J Food Compos Anal. 2011;24:506–15. 10.1016/j.jfca.2011.01.020.

[19] Herrera-Rocha KM, Rocha-Guzmán NE, Gallegos-Infante JA, González-Laredo RF, Larrosa-Pérez M, Moreno-Jiménez MR. Phenolic acids and flavonoids in acetonic extract from quince (Cydonia oblonga Mill.): Nutraceuticals with antioxidant and anti-inflammatory potential. Molecules. 2022;27:2462. 10.3390/molecules27082462. 10.3390/molecules27082462 PMC9027093 35458657

[20] Ranjha MMAN, Amjad S, Ashraf S, Khawar L, Safdar MN, Jabbar S, Nadeem M, Mahmood S, Murtaza MA. Extraction of Polyphenols from Apple and Pomegranate Peels Employing Different Extraction Techniques for the Development of Functional Date Bars. Int J Fruit Sci. 2020;20:S1201–21. 10.1080/15538362.2020.1782804.

[21] Aita S, Capriotti A, Cavaliere C, Cerrato A, Giannelli Moneta B, Montone C, Piovesana S, Laganà A. Andean Blueberry of the Genus Disterigma: A High-Resolution Mass Spectrometric Approach for the Comprehensive Characterization of Phenolic Compounds. Separations. 2021;8:58. 10.3390/separations8050058.

[22] Cerrato A, Piovesana S, Aita SE, Cavaliere C, Felletti S, Laganà A, Montone CM, Vargas-de-la-Cruz C, Capriotti AL. Detailed investigation of the composition and transformations of phenolic compounds in fresh and fermented Vaccinium floribundum berry extracts by high-resolution mass spectrometry and bioinformatics. Phytochem Anal. 2022;33:507–16. 10.1002/pca.3105. 35064611 10.1002/pca.3105 PMC9543071

[23] Cerrato A, Biancolillo A, Cannazza G, Cavaliere C, Citti C, Laganà A, Marini F, Montanari M, Montone CM, Paris R, Virzì N, Capriotti AL. Untargeted cannabinomics reveals the chemical differentiation of industrial hemp based on the cultivar and the geographical field location. Anal Chim Acta. 2023;1278: 341716. 10.1016/j.aca.2023.341716. 37709459 10.1016/j.aca.2023.341716

[24] Ford L, Stratakos AC, Theodoridou K, Dick JTA, Sheldrake GN, Linton M, Corcionivoschi N, Walsh PJ. Polyphenols from Brown Seaweeds as a Potential Antimicrobial Agent in Animal Feeds. ACS Omega. 2020;5:9093–103. 10.1021/acsomega.9b03687. 32363261 10.1021/acsomega.9b03687 PMC7191560

[25] Yang H, Yang C, Sun T. Characterization of glycopeptides using a stepped higher-energy C-trap dissociation approach on a hybrid quadrupole orbitrap. Rapid Commun Mass Spectrom. 2018;32:1353–62. 10.1002/rcm.8191. 29873418 10.1002/rcm.8191

[26] Generalić Mekinić I, Skroza D, Šimat V, Hamed I, Čagalj M, Popović Perković Z. Phenolic Content of Brown Algae (Pheophyceae) Species: Extraction, Identification, and Quantification. Biomolecules. 2019;9:244. 10.3390/biom9060244. 31234538 10.3390/biom9060244 PMC6628088

[27] Gómez-Guzmán M, Rodríguez-Nogales A, Algieri F, Gálvez J. Potential Role of Seaweed Polyphenols in Cardiovascular-Associated Disorders. Mar Drugs. 2018;16:250. 10.3390/md16080250. 30060542 10.3390/md16080250 PMC6117645

[28] Abdelshafeek KA, El-Shamy AM. Review on glucosinolates: Unveiling their potential applications as drug discovery leads in extraction, isolation, biosynthesis, biological activity, and corrosion protection. Food Biosci. 2023;56: 103071. 10.1016/j.fbio.2023.103071.

[29] Schymanski EL, Jeon J, Gulde R, Fenner K, Ruff M, Singer HP, Hollender J. Identifying Small Molecules via High Resolution Mass Spectrometry: Communicating Confidence. Environ Sci Technol. 2014;48:2097–8. 10.1021/es5002105. 24476540 10.1021/es5002105

[30] Rajanikar RV, Nataraj BH, Naithani H, Ali SA, Panjagari NR, Behare PV. Phenyllactic acid: A green compound for food biopreservation. Food Control. 2021;128: 108184. 10.1016/j.foodcont.2021.108184.

[31] Zhao J, Zeng H, Cheng X. Bond dissociation energies for removal of the hydroxyl group in some alcohols from quantum chemical calculations. Int J Quantum Chem. 2012;112:665–71. 10.1002/qua.23037.

[32] Shrestha S, Zhang W, Smid SD. Phlorotannins: A review on biosynthesis, chemistry and bioactivity. Food Biosci. 2021;39: 100832. 10.1016/j.fbio.2020.100832.

[33] Drobnitch ST, Jensen KH, Prentice P, Pittermann J. Convergent evolution of vascular optimization in kelp (Laminariales). Proc R Soc B Biol Sci. 2015;282:20151667. 10.1098/rspb.2015.1667. 10.1098/rspb.2015.1667 PMC4614777 26423844

