# Supplementary material for: Comprehensive profiling of sulfated phenolic compounds in edible and infesting seaweeds by a dedicated software-assisted platform
Source: Anal Bioanal Chem. 2025 Aug 28;417(23):5199–209. doi: 10.1007/s00216-025-06027-3 (PMC12432060; doi:10.1007/s00216-025-06027-3)
Supplement: Supplementary file 1 — Supplementary Material 1 (DOCX 1.06 MB) [file 216_2025_6027_MOESM1_ESM.docx]

**Supplementary Material**

**Comprehensive profiling of sulfated phenolic compounds in edible and infesting seaweeds by a dedicated software-assisted platform**

Enrico Taglioni^1^, Chiara Cavaliere^1^, Andrea Cerrato^1^, Aldo Laganà^1^, Carmela Maria Montone^1,*^, Anna Laura Capriotti^1^

^1^ Department of Chemistry, Sapienza University of Rome, Piazzale Aldo Moro 5, 00185 Rome, Italy

***Corresponding author**

Dr. Carmela Maria Montone

E-mail: carmelamaria.montone@uniroma1.it

tel: +39 06 4991 3945


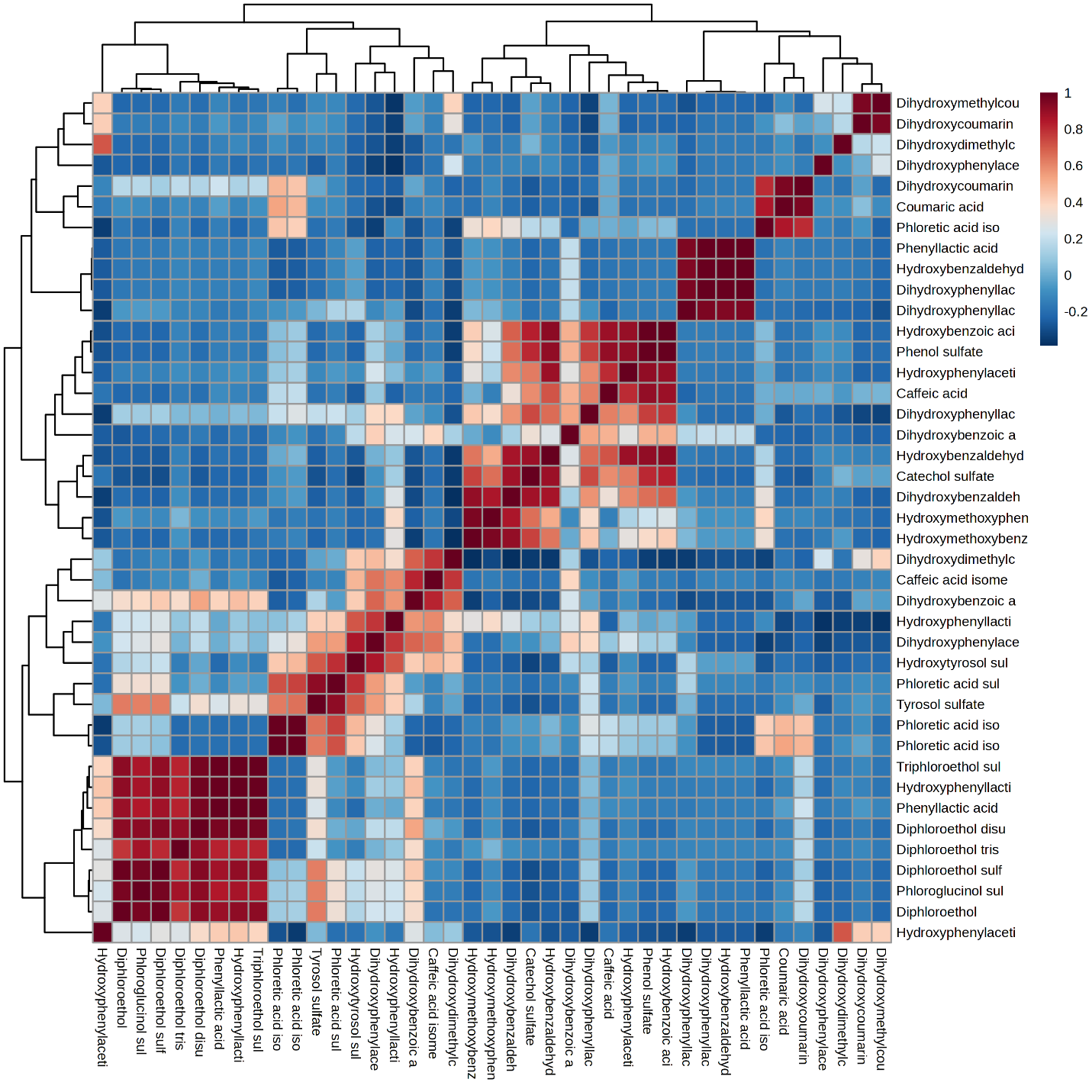


**Figure S1.** Correlation heatmap of the peak areas of the 44 annotated phenolic compounds in the 10 analyzed seaweed strains.


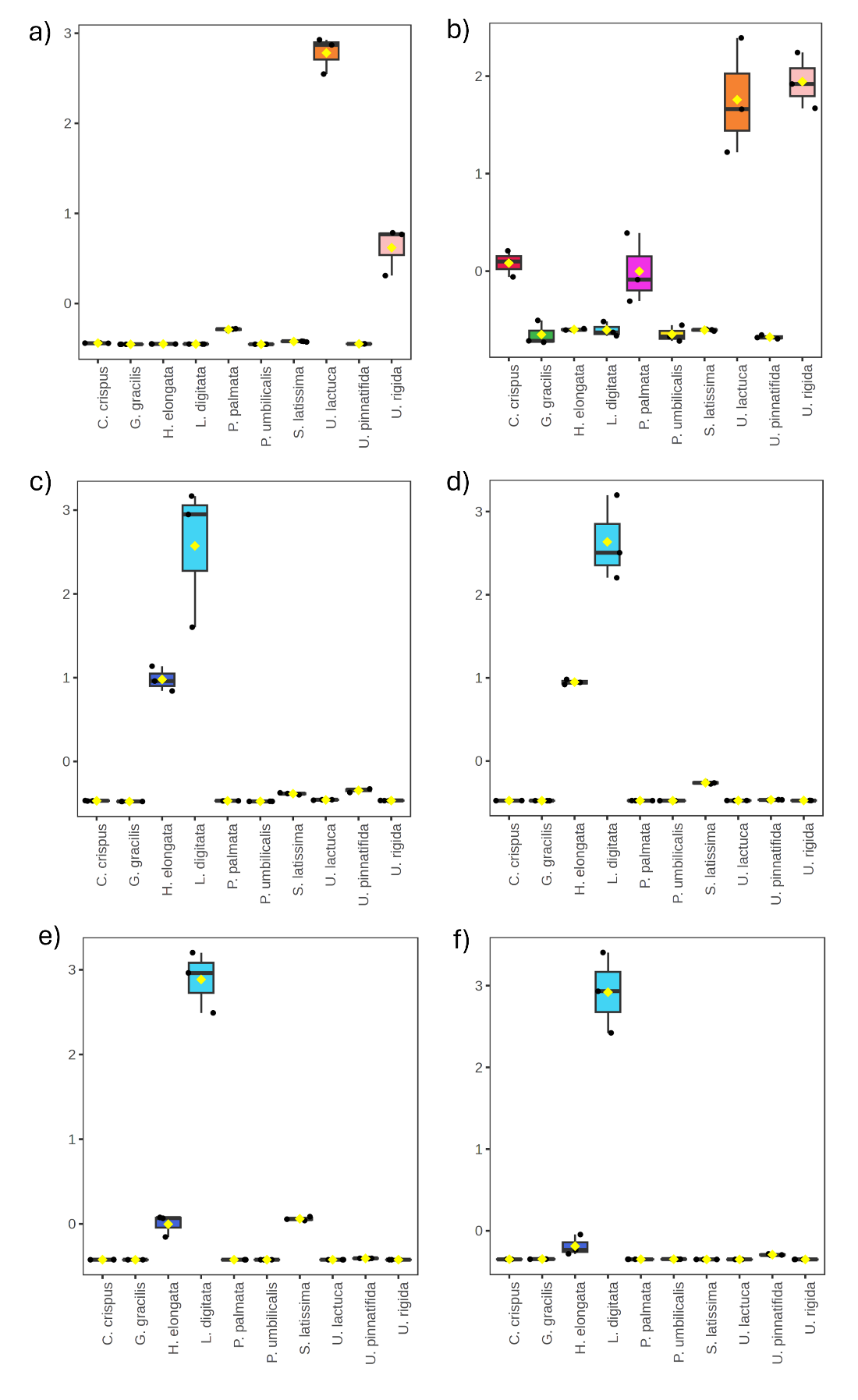


**Figure S2.** Box and whiskers plot of the peak areas of (a) phenol sulfate, (b) catechol sulfate, (c) phloroglucinol sulfate, (d) diphloroethol sulfate, (e) diphloroethol disulfate, and (f) triphloroethol sulfate in the ten analyzed seaweed samples.


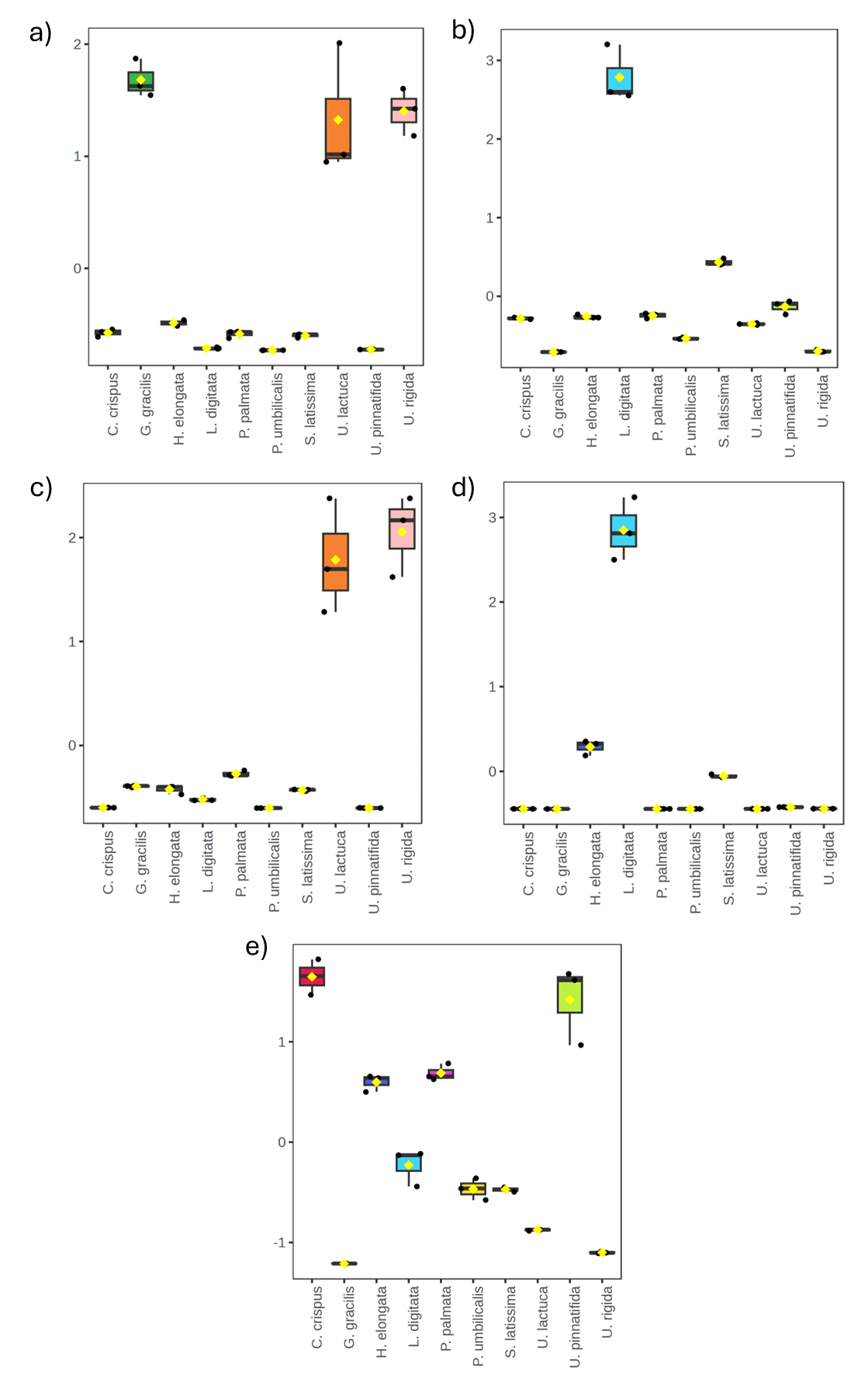


**Figure S3.** Box and whiskers plot of the peak areas of (a) phenol sulfates, (b) phenolic acids, (c) phenolic acid sulfates, (d) phlorotannins, and (e) coumarins in the ten analyzed seaweed samples.


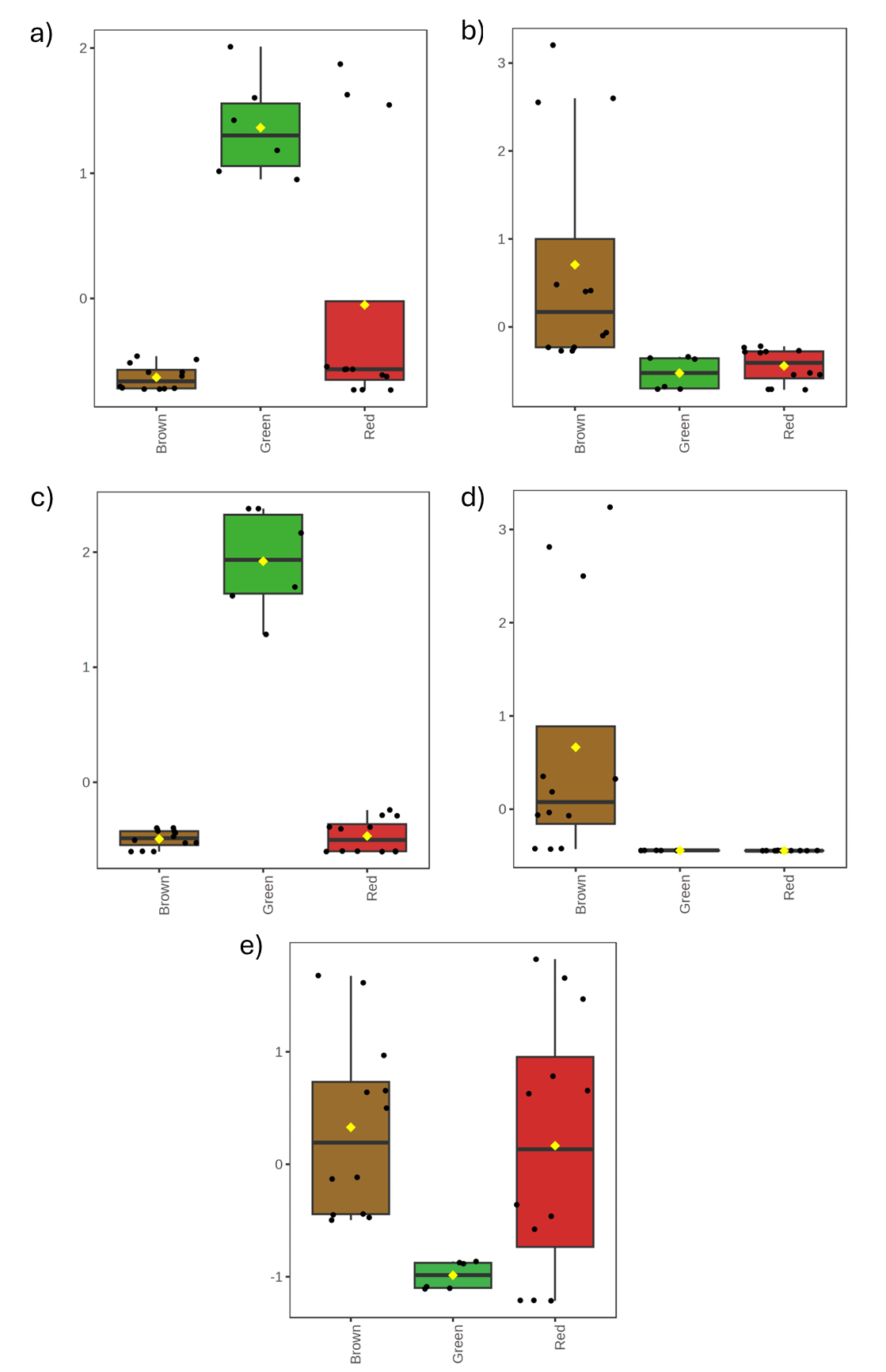


**Figure S4.** Box and whiskers plot of the peak areas of (a) phenol sulfates, (b) phenolic acids, (c) phenolic acid sulfates, (d) phlorotannins, and (e) coumarins in the ten analyzed seaweed samples grouped into three classes based on the taxonomy.
